# Polyhydroxyalkanoate Production from Fruit and Vegetable Waste Processing

**DOI:** 10.3390/polym14245529

**Published:** 2022-12-16

**Authors:** Paolo Costa, Marina Basaglia, Sergio Casella, Lorenzo Favaro

**Affiliations:** Department of Agronomy, Food, Natural Resources, Animals and Environment (DAFNAE), University of Padova, Agripolis, 35020 Legnaro, (PD), Italy

**Keywords:** *Cupriavidus necator*, *Hydrogenophaga pseudoflava*, industrial fruit processing melon and red apple waste, PHAs

## Abstract

Traditional plastics represent a tremendous threat to the environment because of increases in polluting manufacturing as well as their very extended degradation time. Polyhydroxyalkanoates (PHAs) are polymers with similar performance to plastic but are compostable and synthesizable from renewable sources and therefore could be a replacement for fossil-based plastics. However, their production costs are still too high, thus demanding the investigation of new and cheap substrates. In this sense, agricultural wastes are attractive because they are inexpensive and largely available. Specifically, fruit and vegetables are rich in sugars that could be fermented into PHAs. In this work two strains, *Cupriavidus necator* DSM 545 and *Hydrogenophaga pseudoflava* DSM 1034, well-known PHA-producing microbes, were screened for their ability to grow and accumulate PHAs. Ten different fruit and vegetable processing waste streams, never before reported in combination with these strains, were tested. Residues from red apple and melon were found to be the most suitable feedstocks for PHA production. Under specific selected conditions, *C. necator* DSM 545 accumulated up to 7.4 and 4.3 g/L of 3-hydroxybutyrate (3HB) from red apple and melon, respectively. Copolymer production was also obtained from melon. These results confirm the attractiveness of food processing waste as a promising candidate for PHA production. Ultimately, these novel substrates draw attention for future studies on process optimization and upscaling with *C. necator*.

## 1. Introduction

Globally, plastic production grew 20-fold in the last 50 years. In 2018, it accounted for 360 Mt, an amount that might triple by 2050 [1]. A ton of plastic incorporates 2.7 tCO_2_, while 2.5 tCO_2_ are generated during its manufacture [2]. In addition, since they degrade very slowly under environmental conditions, conventional fossil-based plastics massively pollute the environment for a prolonged time [3]. As a promising alternative, polyhydroxyalkanoates (PHAs) represent a class of compostable biopolymers that can be produced from renewable sources. Furthermore, PHAs show physical properties as excellent as those of traditional plastics, thus attracting the attention of society and industry [4]. PHAs indeed find potential use in several sectors, including agro-industry, packaging, therapeutic practices, surgical implants, and building blocks [5]. Several prokaryotes accumulate PHAs in the form of intracellular granules as energy storage and as a barrier that provides robustness and higher stress survival [6]. Cells synthesize PHAs from fatty acids or sugars, especially under nitrogen-deficient conditions [7,8]. *Cupriavidus necator* is one of the most studied PHA-producing bacteria, being able to accumulate up to 90% of its total cell dried weight [9]. At the end of the fermentation process, cells are disrupted and PHA granules purified through a solvent extraction process [10].

In 2019, PHAs accounted for 1.2% of global bioplastic production and are expected to increase to 3.8% by 2024 [11]. However, the size of the bioplastic market is still incomparable with the one for traditional plastics: in 2021 it represented less than 1% of global plastic production [12]. To make PHAs more competitive in the market, the production price needs to decrease. Above all, substrates for growth, generally pure sugars or fatty acids, represent 50% of the costs alone [9,13]. It is thus necessary to search for novel and low-cost carbon-rich substrates. Agricultural wastes like fruit and vegetables removed from the supply chain during cleaning, processing and packaging procedures might represent an attractive candidate. These wastes are mostly composed of leaves, peels, pomace, skins, rinds, core, pits, pulps, stems, seeds, and twigs, rich in sugars and nutrients potentially useful for bacterial growth [14]. Because of its renewable nature, large availability, and its reduced cost, food waste has been gaining more and more attention, and it is no coincidence that the number of scientific papers regarding its exploitation grew exponentially in the last decade [15,16,17,18,19,20,21,22,23].

The Food and Agriculture Organization (FAO) estimates that one-third of food produced is disposed of worldwide [24]. In addition, despite optimization of the supply chain and refined recycling processes, a portion of food is always unavoidably wasted [15]. As a matter of fact, the use of agricultural wastes for PHA production would not affect the food supply and human nutrition [25]. Additionally, food waste represents not only an abundant and underestimated resource but also an environmental burden to dispose of. Greenhouse gases are emitted during landfill disposal, manufacturing, transportation and storage, including 3.49 billion tCO_2_ [26]. Economically, the FAO calculated that overall food waste could correspond to a 490 billion USD loss per year [27].

Conventional waste management, which includes landfilling, composting, animal feeding, and thermal treatment, must thus be flanked by innovative and more profitable valorization routes such as PHA production. However, despite being abundant and renewable, wastes are also complex biomasses. To address an industrial process, these novel substrates should be homogenous and characterized by constant qualitative and quantitative standards over time [9,28]. In fact, small biorefineries processing local feedstocks generated from industry are more reliable [20,29,30,31].

Therefore, PHA production from agricultural waste may represent an encouraging solution. One first example is provided by the employment of chicory roots as a substrate for PHB production by three different strains of *C. necator* [32]. In another paper, Haas et al. took advantage of the high starch concentration of potatoes waste as a carbon source for *C. necator* PHA accumulation [33]. Sweet potato by-products and broken rice were tested by Brojanigo et al. with successful outcomes: 3HB was produced up to 5.18 g/L [34]. It is of note that broken rice was then processed into PHB through a novel consolidated bioprocessing approach using an engineered *C. necator* amylolytic strain [35]. Since lipids are also suitable for the biosynthesis of PHA, olive oil and slaughterhouse wastes might represent interesting food waste sources [36,37]. Waste rapeseed oil was indeed converted into PHAs by *C. necator* in a fed-batch process with the productivity of 1.46 g/L/h [38]. Finally, fruit wastes also showed a promising performance when used as a substrate: apricots, cherries, and grapes were successfully converted by *Pseudomonas resinovorans* into a blend of *mcl*-PHAs, and the maximum yield accounted for 21.3 g/L of substrate [39].

In this work, for the first time ten different fruit and vegetable processing by-products widely available in Italy [40] were investigated as possible feedstocks for *C. necator* and *Hydrogenophaga pseudoflava* DSM 1034, another well-studied strain able to accumulate polymers from a wide range of substrates. The wastes, including both fruits and vegetables, were collected, processed, and pre-treated through a thermal extraction process, and their chemical components analyzed. The goal of this study was to evaluate, starting from novel substrates for the two strains, the growth under batch conditions and the accumulation of short-chain PHAs such as 3-hydroxybutyrate and 3-hydroxyvalerate (3HB and 3HV). Furthermore, different strategies were considered to investigate the substrate pre-treatment and sterilization. Ultimately, the chemical composition of the medium was tailored to boost PHA production from the selected by-products.

## 2. Materials and Methods

### 2.1. Bacterial Strains, Agricultural Residues, and Preparation, Treatment, and Analysis

*C. necator* DSM 545 and *H. pseudoflava* DSM 1034, supplied by DSMZ (Braunschweig, Germany), were employed as PHA-producing strains, maintained at −80 °C in a 25% glycerol solution and plated on nutrient agar Petri dishes when needed (g/L: peptone 15, yeast extract 3, NaCl 6, glucose 1, agar 15).

The preliminary set of agricultural wastes tested originated from a capillary availability assessment of inexpensive agricultural residues from northern Italy [40]. Briefly, samples of about 10 Kg of fruits and vegetables had been collected in an area of 11,300 Km^2^ with its center in the municipality of Soave (Province of Verona, Italy, latitude: 45°25′10″ N, longitude: 11°14′45″ E). The fruits had undergone a freeze-drying step, while the vegetables had been dried at 60 °C in a forced-air oven for 48 h. All of them had eventually been ground in a hammer mill through a 1.0 mm sieve. From more than 80 wastes, only 10 were selected for this work, specifically only the ones that lacked any literature regarding their conversion to PHAs mediated by *C. necator* and *H. pseudoflava*. This set included wastes of cucumber, peach, onion, plum, and melon, as well as industrial wastes of apricot, yellow apple, pear, tomato, and red apple. Furthermore, a second group of fruits were processed that included wastes from red apples of the variety Red Delicious (Marlene^®^) and melons of the cantaloupe variety provided by a local fruit supplier.

The dry matter, protein, starch, hemicellulose, cellulose, and lignin for every waste product were measured following international standard methodologies: total ash and moisture were acquired, respectively, by calcinating the residues at 550 °C, which were then dried in a oven at 103 °C, as the Association of Official Analytical Chemists (AOAC) guidelines indicate (methods 942.05 and 934.01) [41]. Cellulose, hemicellulose, and lignin were measured according to Van Soest et al. methodology [42]. Starch contents were calculated according to AOAC method 920.40. Successively, nutrients were extracted from the waste through a water extraction process. For 1 g of dried or freeze-dried biomass, 20 mL of distilled water were added. Extractions occurred at 85 °C on a magnetic stirrer for 45 min. Later, solutions were centrifuged for 7 min at 6000× *g* at 10 °C. Liquid upper phases were re-collected and filtered through 0.2 µm sterile filters. Differently, solutions from the Red Delicious and cantaloupe extracted after centrifugation were either filtered or autoclaved (121 °C, 20 min).

### 2.2. Culture Media and Experimental Set-Up

Inocula of *C. necator* DSM 545 and *H. pseudoflava* DSM 1034 were performed at 30 °C in 100 mL flasks under aerobic conditions (145× *g*) with a final volume of 30 mL. DSMZ-81 culture medium was prepared at 10 times concentration and supplemented with 30 g/L D-glucose. The DSMZ-81 broth had the following composition (g/L): NH_4_Cl 1, MgSO_4_·7H_2_O 0.5, NaHCO_3_ 0.5, KH_2_PO_4_ 2.3, Na_2_HPO_4_·7H_2_O 2.9, CaCl_2_·2H_2_O 0.01, and ferric ammonium citrate 0.05. Afterward, in 1 L of DMSZ-81 medium, 0.5 mL of microelement solutions were added. The composition of the latter mixture was the following (g/L): ZnSO_4_·7H_2_O 0.1, MnCl_2_·4H_2_O 0.03, H_3_BO_3_ 0.3, CoCl_2_·6H_2_O 0.2, CuCl_2_·2H_2_O 0.01, NiCl_2_·6H_2_O 0.02, and Na_2_MoO_4_·2H_2_O 0.03. DMSZ-81 was also supplemented with the following standard vitamin solution (final concentration mg/L): riboflavin 0.0005; thiamine-HCl·2H_2_O 0.0025; nicotinic acid 0.0025; pyridoxine-HCl 0.0025; Ca-pantothenate 0.0025; biotin 0.000005; folic acid 0.00001; and vitamin B12 0.00005. After overnight growth, bacterial cells were harvested by centrifugation (6000× *g* for 15 min), washed twice, and resuspended in the same volume of NaCl 0.9% (*w*/*v*).

Initially, all the waste-derived extracted solutions, enriched with 10-fold DSMZ-81 medium (10% of total volume), were screened as possible substrates for *C. necator* DSM 545 and *H. pseudoflava* DSM 1034 growths. Thus, cells were aerobically inoculated at 30 °C under shaking for 66 h onto a 96-well plate loaded on a TECAN SPARK 10M reader monitoring bacterial growth by measuring optical density (OD_600_ nm) every hour. After that, the experiment was replicated on a larger scale, in 100 mL flasks, up to a working volume of 30 mL, replicating the conditions described for the experiment with TECAN technology, although fermentations were extended up to 96 h. Since the DMSZ-81 medium was ten times more concentrated to minimize substrate dilution, 27 mL of extracted waste solution was used for each flask and enriched with 3 mL of concentrated DSMZ-81.

Besides, with pear, tomato, Red Delicious apples and Cantaloupe melons three further conditions were tested on 100 mL flasks to investigate the relationship between nitrogen presence and PHAs accumulation. Specifically, the growth medium was composed either of distilled water or DSMZ-81 or DSMZ-81 modified by substituting NH_4_Cl with NaCl. Furtherly, only for Red Delicious apples and Cantaloupe melons the sterilization strategy was evaluated: the wastes-derived solutions were either filtered or autoclaved.

Experiments were performed in triplicate and standard deviation is reported.

### 2.3. PHA Analysis

Firstly, after growing for 96 h the cells were pelleted for 15 min at 6000× *g*, stored at −20 °C, and lyophilized overnight. Then, cell dried matter (CDM) was measured. 3-hydroxybutyric acid (3HB) and 3-hydroxyvalerate acid (3HV) monomers were analyzed according to Torri and colleagues [43] and, mostly, after their extraction [44] by gas chromatography using a Thermo Finnigan Trace GC equipped with a flame ionization detector (FID) and AT-WAX column (30 m × 0.25 mm × 0.25 μm). The gas carrier was helium at a flow rate of 1.2 mL/min, and the split/splitless injector with a split ratio 1:30 was set at 250 °C. FID and oven temperature were set at 270 and 150 °C, respectively. Benzoic acid was used as internal standard, whereas the external standards 3HB, poly (3-hydroxybutyric acid-co-3-hydroxyvaleric acid), and P(3HB-co-14 mol% 3HV) were purchased from Sigma-Aldrich (Milan, Italy).

Results were reported as the percentage of PHAs in CDM or grams of PHAs/liter of culture.

### 2.4. Analytic Methodologies

Glucose, fructose, cellobiose, and sucrose measurements were performed through high-performance liquid chromatography (HPLC) using a Shimadzu Nexera HPLC system equipped with a RID-10A refractive index detector (Shimadzu, Kyoto, Japan) [45]. The chromatographic separations were performed using a Phenomenex Rezex ROA-Organic Acid H+ (8%) column (300 mm × 7.8 mm). The column temperature was set at 60 °C, and the analysis was performed at a flow rate of 0.6 mL/min using isocratic elution, with 5 mM H_2_SO_4_ as mobile phase. Analytes were identified by comparing their retention times, and the concentrations were calculated using the calibration curve of the corresponding external standard. Finally, total nitrogen and phosphate content were measured using the Kjeldahl methodology [46] and total polyphenols were estimated by Folin–Ciocâlteu assay [47].

### 2.5. Statistical Analysis

All results, including optical density (OD_600_), cell dried matters (CDM), and PHA concentration values originate from experiments run with three biological replicates. Standard deviations and *t*-test for significance were calculated using Microsoft Excel.

## 3. Results and Discussion

### 3.1. Chemical Composition and Sugar Extraction from Fruit and Vegetable Wastes

Since *C. necator* and *H. pseudoflava* grow largely on glucose and fructose, this work specifically targets agricultural wastes rich in reducing sugars that are widely produced in Italy. The dried matter, cellulose, hemicellulose, lignin, starch, and protein contents of the waste material studied are reported in Table 1. The highest percentage of dried matter (30.3%) was recorded for pear, while cucumber and melon accounted for only 5.7 and 6.7%, respectively. Looking at fermentable sugars, starch was detected only in low amounts in cucumber, onion, and tomato (0.3, 0.3, and 0.7%, respectively). Concerning cellulose, while apple and onion account for the lowest content (6.0, 5.4, and 6.2%, respectively), pear waste scores the highest (30.2%). Similarly, hemicellulose is particularly low in the case of onion and apple, while very highly concentrated in pear wastes. A possible explanation involves the origin of pear wastes: since they are by-products of pulp machines, peel rich in cellulose and hemicellulose is likely the main fraction. This explanation also supports the high result in terms of lignin content for tomato waste (31.0%), as this feedstock is also derived from a pulping machine. However, while cellulose and hemicellulose may be converted into fermentable sugars upon hydrolysis, lignin is not a fermentable polymer. Finally, melon, tomato, and cucumber had the greatest amount of protein (16.7, 18.2, and 14.8%, respectively) while on the other hand apple and pear had the smallest shares (2.9 and 3.1%, respectively).

An extraction performed in water at high temperature was conducted to recover nutrients from the wastes. Specifically, as reported in Table 2, solutions originating from red and yellow apples accounted for the highest concentration of soluble sugars (36.54 and 31.21 g/L, respectively) resembling, as in the case of apricot, nectarine, and onion, the composition of the fruits of origin [48,49,50,51]. On the other hand, from tomatoes and pears, a very small amount of sugars was extracted (1.6 and 0.83 g/L, respectively). Regarding total nitrogen and phosphorous, very few surveys were found in the literature. Specifically, regarding nitrogen, nectarine recorded the largest concentration (0.77 g/L), even more than expected [50], while tomato had the lowest (0.01 g/L). Looking at total phosphorous, the extraction showed a scenario close to the one for nitrogen: nectarine waste had the highest concentration (0.31 g/L), which is similar to that of the raw fruit [49], while tomato, red apple, and melon had the lowest (0.02 and 0.03 g/L). Ultimately, total polyphenol concentrations were also measured, and the largest fraction (about 1 g/L) was extracted from cucumber and onion, while the smallest ones were from tomato and pear (0.2 and 0.17 g/L, respectively), although still in line with scientific evidence [51,52].

### 3.2. Initial Screening of C. necator DSM 545 and H. pseudoflava DSM 1034 Growth with Different Agricultural Wastes

**Figure 1:**
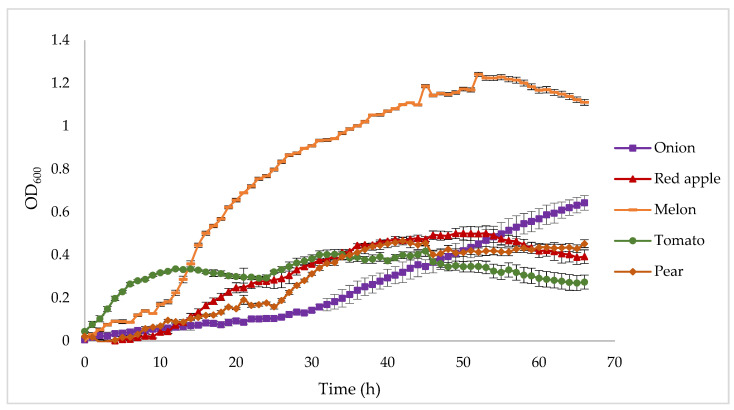
Growth (OD_600_) of *C. necator* DSM 545 at 30 °C in DMSZ-81 broth supplemented with water-extracted solutions of different fruit and vegetable waste. The data are the means of three replicates (±SD).

A preliminary selection of possible suitable wastes employable for PHA production by *C. necator* DSM 545 and *H. pseudoflava* DSM 1034 was performed firstly on a micro-scale level. Using TECAN technology, growth curves for each agricultural waste were obtained (Figure 1). *H. pseudoflava* DSM 1034 showed consistent growth for none of the tested wastes. OD_600_ values never overcame 0.2, indicating that the selected substrates are poor candidates to support PHA production by *H. pseudoflava* DSM 1034 (data not shown). Similarly, *C. necator* DSM 545 grew only faintly on some of the candidate substrates, such as apricot, cucumber, nectarine, plum, and yellow apple. For this reason, the latter wastes and *H. pseudoflava* were excluded from following screenings. Onion, red apple, tomato, pear, and melon appeared as potential novel substrates for *C. necator* DSM 545 growth (Figure 1). After 66 h, with the only exception for onion, the growth kinetics showed that the exponential phase was completed for all the mentioned wastes. Melon appeared as the most promising substrate since OD_600_ reached a value of 1.24, suggesting biomass production larger than those obtained from the other residues.

**Figure 2:**
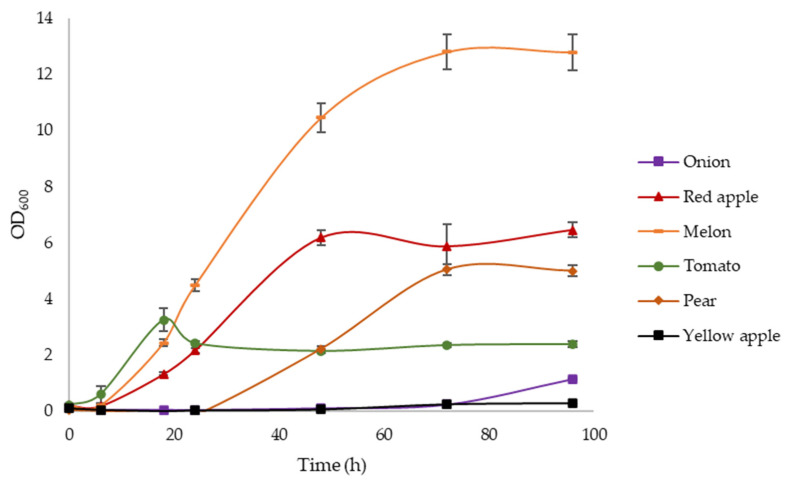
Growth (OD_600_) of *C. necator* DSM 545 at 30 °C for 96 h in flasks in DMSZ-81 broth supplemented with water-extracted solutions of different fruit and vegetable waste. The data are the means of three replicates (±SD).

To confirm the results achieved at small scale, the experiment was replicated on larger volumes, in flasks. This time, only wastes showing promising outcomes during the first screening were tested, adding yellow apple as a negative control. As summarized in Figure 2, melon was again the best substrate (max OD_600_ = 12.8), followed by red apple. Biomass highly developed on tomato and pear as well, though at a lower rate; meanwhile, differently from what was assessed previously, no growth was observed when *C. necator* DSM 545 was incubated with onion, perhaps because of the well-known content of antimicrobial compounds naturally present in onion extract [53]. Lastly, yellow apple appeared again as a weak potential substrate for *C. necator* DSM545, overall confirming the results of the first screening and the consistency of the methodology (Figure 1). From these results, it is difficult to point out a correlation between the substrate composition and the relative biomass production. For instance, it is interesting to notice how differently *C. necator* DSM 545 behaves when grown on red or yellow apples: although their compositions are very similar in terms of sugars, they quite differ in their contents of nitrogen, phosphorous, and especially polyphenols. This latter category would require some deeper investigation to address which specific polyphenols might trigger or curb *C. necator* DSM 545 growth.

### 3.3. PHA Production from Selected Agricultural Wastes

Tomato, pear, red apple, and melon were then selected to be employed as substrates for PHA production by *C. necator* DSM 545. Three broth conditions were adopted: the solutions originating from the waste extraction in water were enriched either with DMSZ-81 culture medium, DMSZ-81 deprived of its nitrogen source (NH_4_Cl substituted equimolarly with NaCl), or distilled water. Thus, it was possible to investigate how much both the waste alone (once supplemented in water) and the nutrients from DSMZ-81 are relevant for *C. necator* DSM 545 growth and PHA accumulation. Specifically, by removing NH_4_Cl from DMSZ-81 or by using water, it was possible to address whether the stress generated by nitrogen limitation had an impact on cell growth and PHA accumulation.

As displayed in Table 3, biomass was not produced when only water was added for both tomato and pear, proving that nutrients from DSMZ-81 broth are essential for bacterial growth. Interestingly, complete DMSZ-81 broth supported biomass production but had a negative effect on PHA accumulation, which instead was greatly improved when the ammonium chloride was removed (up to 54.5% of CDM). This finding may be explained by considering that the two waste solutions already contained amounts of nitrogen (Table 2) limited enough to trigger PHA production but lacked the other nutrients for cell growth.

When it came to melon and red apple, new, fresh batches of fruits were tested. Notably, 3HV (3-hydorxy-valerate) was detected as a final product. In addition, two different strategies for sterilization were used: either filtration, as for tomato and pear, or autoclaving. In fact, filtration is indeed a very expensive and time-consuming technique, and autoclaving might offer a fast and inexpensive strategy. As reported in Table 3, if compared with tomato and pear, red apple and melon gave better yields in terms of both cell dried matter (up to 10.9 g/L) and 3HB accumulation (up to 79.7%). Contrary to the cases of tomato and pear, if only water was added, with the only exception of the autoclaved melon, growth and 3HB production still occurred significantly (up to 3.9 g/L 3HB). Considering the sterilization strategy, when melon was used, autoclaving triggered 3HB accumulation and elicited 3HV production (up to 0.23 g/L). This is most likely due to the release under autoclaving conditions of molecules able to act as 3HV precursors for *C. necator* DSM 545. On the other hand, regarding red apple a reduced 3HB accumulation can be observed when autoclaving was adopted as the sterilization method instead of filtration (maximum 30.8%). Overall, the best-performing tested condition was provided by the use of red apple filtered and enriched with DMSZ-81 (7.4 g/L 3HB).

When compared to the literature, the biomass and PHA yields obtained in this study are of great interest (Table 4). For several research works, different strains of *C. necator* were grown, using a batch strategy, with the purpose of PHB accumulation. Nevertheless, the detection of copolymers occurs quite rarely, although it represents an important feature since the material has superior physical properties [54]. In this sense, melon, tested in this work, becomes a very attractive waste: its conversion into 3HV yielded 4.6% of cell dried matter. Therefore, differently from red apple, it is reasonable to infer that it holds precursors for 3HV production, potentially released, as already discussed above, after autoclaving. Similarly, brewery wastewater was found as a promising feedstock for co-3HV production, accounting for a higher content (11.5% of CDM) although the overall production of PHAs represented only 38% of total biomass [55]. Significantly, both for melon and red apple, PHB accumulation recorded notable titers, larger than in all the reported works. Tested in flasks, banana and pineapple extracts were studied by other groups [56,57]. While in the case of banana, *C. necator* was able to grow and accumulate PHB less than in the present study (3.6 g/L biomass and 37.4% PHB content), pineapple showed remarkable values: final biomass reached up to 13.6 g/L with an intracellular concentration of 60% PHB. Overall, the highest biomass yield was obtained adopting *C. necator* (strain NCIMB 11599) on wheat bran hydrolysate [58]. Nevertheless, high enzyme loading as well as energy-intensive processes were needed to achieve such high cell titers, whereas this study specifically made use of water extraction as a low-cost substrate pretreatment.

## 4. Conclusions

The goal of this study was the search for novel low-cost substrates to produce PHAs using *Cuprividus necator* 545 and *Hydrogenophaga pseudoflava* 1034. Here, some agricultural wastes, especially red apple and melon, gave promising performances for possible future applications. By exploiting a simple and inexpensive extraction in water, it was possible, even using different batches of fruits, to convert very cheap substrates into high-value 3HB-co-3HV. *C. necator* DSM 545 was able to produce a significantly large biomass and accumulate extensive amounts of 3HB, up to 7.4 g/L. In the future, the processes need to be scaled up and tested under different strategies, including fed-batch fermentation. Additionally, fresh wastes from large suppliers undergoing shorter and more simple pretreatments might be tested, in view of a real-world scenario.

## Figures and Tables

**Table 1:** Chemical content (% of dried matter) of agricultural wastes considered in this work. Wastes labeled by * originated from industrial processes.

(% Dry Mass)						
Composition	Dry Mass (%)	Cellulose	Hemicellulose	Lignin	Starch	Protein
Cucumber	5.70	16.30	5.60	1.80	0.30	18.20
Nectarine	20.80	17.60	9.20	12.60	-	7.20
Onion	11.10	6.20	2.50	0.40	0.30	10.90
Plum	14.50	15.70	7.30	10.20	0.00	6.40
Melon	6.70	18.30	3.50	5.60	-	14.80
Apricot pomace *	17.0	16.00	7.10	10.10	-	7.20
Yellow apple *	11.10	6.00	3.10	3.80	-	2.90
Pear *	30.30	30.20	25.60	20.40	-	3.10
Tomato *	21.80	16.40	10.50	31.00	0.70	16.70
Red apple *	12.60	5.40	2.90	2.80	-	2.90

**Table 2:** Composition (g/L) of extracted solutions for the agricultural wastes considered in this work. Wastes labeled by * originated from industrial processes. nd: not detected.

Waste	Glucose	Fructose	Cellobiose	Total Sugars	Total Nitrogen	Total Phosphorus	Total Polyphenols
Cucumber	1.63	6.26	nd	7.89	0.45	0.20	1.07
Nectarine	5.63	6.48	nd	12.11	0.77	0.31	0.40
Onion	12.35	10.72	nd	23.07	0.15	0.06	0.97
Plum	11.86	8.24	0.41	20.51	0.58	0.16	0.57
Melon	3.06	7.14	nd	10.20	0.09	0.03	0.45
Apricot pomace *	5.30	3.49	nd	8.79	0.15	0.07	0.45
Yellow apple *	10.29	19.40	1.52	31.21	0.17	0.18	0.43
Pear *	0.17	0.66	nd	0.83	0.20	0.06	0.17
Tomato *	0.67	0.93	nd	1.60	0.01	0.02	0.20
Red apple *	10.80	22.14	3.60	36.54	0.07	0.02	0.29

**Table 3:** Biomass production and 3HB and 3-hydroxybutyrate (3HV) accumulation by *C. necator* DSM 545 after 96 h from tomato, pear, red apple, or melon as the sole carbon sources. Flasks were supplemented with either complete DSMZ-81, DSMZ-81 deprived of NH_4_Cl, or water. Media were sterilized either by filtering or autoclaving. The values represent the mean of three replicates (±SD).

	Sterilization	Culture Medium	CDM (g/L)	3HB (% CDM)	3HB (g/L)	3HV (% CDM)	3HV (g/L)
		DSMZ-81	0.7 ± 0.22	0.3 ± 0.6	-	-	-
Tomato	Filtration	DSMZ-81 (no NH_4_Cl)	1.3 ± 0.03	34.6 ± 2.5	0.4 ± 0.1	-	-
		Water	0.1 ± 0.00	-	-	-	-
		DSMZ-81	1.2 ± 0.02	1.3 ± 0.7	0	-	-
Pear	Filtration	DSMZ-81 (no NH_4_Cl)	0.8 ± 0.04	54.5 ± 11.3	0.4 ± 0.2	-	-
		Water	0.1 ± 0.01	-	-	-	-
		DMSZ-81	10.9 ± 0.1	67.9 ± 0.6	7.4 ± 0.01	-	-
Red apple	Filtration	DSMZ-81 (no NH_4_Cl)	6.2 ± 0.2	79.1 ± 0.9	4.9 ± 0.03	-	-
		Water	4.9 ± 0.2	79.7 ± 0.1	3.9 ± 0.04	-	-
		DMSZ-81	6.5 ± 0.5	21.6 ± 1.0	1.4 ± 0.1	-	-
Red apple	Autoclaving	DSMZ-81 (no NH_4_Cl)	6.7 ± 0.04	34.5 ± 2.1	2.3 ± 0.1	-	-
		Water	5.9 ± 0.5	30.8 ± 2.4	1.8 ± 0.1	-	-
		DMSZ-81	9.6 ± 0.5	20.3 ± 2.3	1.94 ± 0.1	-	-
Melon	Filtration	DSMZ-81 (no NH_4_Cl)	4.2 ± 0.4	34.0 ± 6.7	1.43 ± 0.2	-	-
		Water	0.3 ± 0.1	-	-	-	-
		DMSZ-81	5.9 ± 0.5	32.6 ± 6.1	1.93 ± 0.2	3.1 ± 0.2	0.18 ± 0.1
Melon	Autoclaving	DSMZ-81 (no NH_4_Cl)	5.8 ± 0.4	73.8 ± 10.1	4.26 ± 0.1	0.7 ± 1.1	0.04 ± 1.7
		Water	5.1 ± 0.3	74. 9 ± 6.6	3.84 ± 0.1	4.6 ± 0.3	0.23 ± 0.1

**Table 4:** Different food waste streams employed to produce 3HB and 3HV from various strains of *C. necator* under a batch fermentation strategy in flasks.

Food Waste	Microorganism	Biomass (g/L)	PHB Content (%)	3HV (%)	Reference
Treated brewery wastewater	*C. necator* DSM 545	7.9	26.5	11.5	[55]
Banana frond extract	*C. necator* H16	3.6	37.4	-	[56]
Crude aqueous extract of pineapple waste	*C. necator* A-04	13.6	60.1	-	[57]
Wheat bran hydrolysate	*C. necator* H16 (mutant NCIMB 11599)	24.43	62.5	-	[58]
Melon extract	*C. necator* DSM 545	5.1	74.9	4.6	This study
Red apple extract	*C. necator* DSM 545	10.9	67.9	-	This study

## Data Availability

Not applicable.
